# Advanced physical techniques for gene delivery based on membrane perforation

**DOI:** 10.1080/10717544.2018.1480674

**Published:** 2018-07-03

**Authors:** Xiaofan Du, Jing Wang, Quan Zhou, Luwei Zhang, Sijia Wang, Zhenxi Zhang, Cuiping Yao

**Affiliations:** Key Laboratory of Biomedical Information Engineering of Ministry of Education, Institute of Biomedical Analytical Technology and Instrumentation, School of Life Science and Technology, Xi’an Jiaotong University, Xi’an, People’s Republic of China

**Keywords:** Gene therapy, electroporation, magnetoporation, sonoporation, optoporation

## Abstract

Gene delivery as a promising and valid tool has been used for treating many serious diseases that conventional drug therapies cannot cure. Due to the advancement of physical technology and nanotechnology, advanced physical gene delivery methods such as electroporation, magnetoporation, sonoporation and optoporation have been extensively developed and are receiving increasing attention, which have the advantages of briefness and nontoxicity. This review introduces the technique detail of membrane perforation, with a brief discussion for future development, with special emphasis on nanoparticles mediated optoporation that have developed as an new alternative transfection technique in the last two decades. In particular, the advanced physical approaches development and new technology are highlighted, which intends to stimulate rapid advancement of perforation techniques, develop new delivery strategies and accelerate application of these techniques in clinic.

## 1. Introduction

Over the past 30 decades, gene-based therapy as a promising and valid tool has been used for treating many serious diseases that conventional drug therapies cannot cure (Ibraheem et al., 2014; Moss, 2014), such as cancer (Johnson et al., 2009), acquired immunodeficiency syndrome (ADIS) (DiGiusto et al., 2010). The main goal of gene therapy is to introduce exogenous nucleic acids or synthetic oligonucleotide into the special cell to treat a disease (Yao et al., 2008; Hao et al., 2014). 

To date, there are many techniques can achieve this goal including biological, chemical, mechanical and physical methods (Mehier-humbert & Guy, 2005; Kim & Eberwine, 2010). Biological method, such as virus-mediated method, is easy to use and with high efficiency (Kim & Eberwine, 2010). But it has potentially risk that viral vectors may infect healthy cell adjacent to the target cell (Moss, 2014), even can lead to patient death because of inflammatory immune responses induced by adenoviral vectors (Chuah et al., 2003), and the size of genetic materials inserted into cell is limited (El-Aneed, 2004; Lv et al., 2006). Chemical methods commonly include calcium phosphate coprecipitation, high molecular weight cationic polymers, cationic lipid and cationic amino acid (Holmen et al., 1995; Washbourne & McAllister, 2002; Kim & Eberwine, 2010; Todorova, 2011). Compared with biological method, the chemical method has advantages of no size limitation and less cytotoxicity. However, the transfection efficiency is lower than biological method (Washbourne & McAllister, 2002; Kim & Eberwine, 2010). For the mechanical methods, such as micro injection (Zhang & Yu, 2008), ballistic (gene gun) (Sun et al., 1998; Trimble et al., 2003), are invasive and cell-damageable. Microinjection is to penetrate the cell membrane with the help of micropipette and deliver the nucleic acids into the cytoplasm (Bora, 2014), which has highly technical and experiential demands for operators. The principle of gene gun is to shot the nucleic acids coated by particles into cell with the help of air pressure (Herrero et al., 2017). However, the air pressure may damage the cell.

Recently, more attentions are focused on physical methods that using physical force to perforate cell membrane and introduce the exogenous nucleic acids into the cell, such as electroporation (Gehl, 2003; Frey et al., 2006; Kalams et al., 2013; Weiland et al., 2013; Schwarz et al., 2018; Markelc et al., 2018), optoporation (Tirlapur & König, 2002; Schneckenburger et al., 2002; Hellman et al., 2008; Dhakal et al., 2015; Batabyal et al., 2017), sonoporation (Liu et al., 2012; Lentacker et al., 2014; Nomikou et al., 2018) and magnetoporation (Li et al., 2008; Chen et al., 2009; Polyakova et al., 2017). As the merit of nontoxicity, these methods are extensively studied and applied in different field, such as biology research (Meacham et al., 2014), tissue engineering (Mellott et al., 2013) and so on. In addition, some of them are noninvasive and non-contact, such as magnetoporation, sonoporation and optoporation. These methods can not only perforate the cell membrane and deliver exogenous nucleic acids, but also directly cure disease. For example, laser with high intensity and long duration pulse can directly kill the tumor cells. With the development of technology, these advanced physical methods will be supposed to become the mainstream technique for gene therapy and gene transfer.

To this day, although a few excellent reviews focused on physical gene delivery techniques are available, they put emphasis on certain applications and the conventional approaches (Mehier-humbert & Guy, 2005; Villemejane & Mir, 2009; Kim & Eberwine, 2010; Mellott et al., 2013; Ibraheem et al., 2014; Hao et al., 2014; Meacham et al., 2014; Jakutavičiūtė et al., 2017; Herrero et al., 2017). In this review, we only pay attention to advanced physical methods and their up-to-date development with a discussion given to the merits and limitations. Moreover, the recent developments in gene therapy are reviewed. Over the past years, nanomaterial technology was rapidly developed and employed to promote these physical approaches for gene delivery. Our team has also devoted to research on the perforating mechanisms and conditions of nanoparticles mediated optoporation for a long time (Yao et al., 2005a, 2008, 2009a). The results were cited by many scholars in their articles (Heinemann et al., 2013; Sengupta et al., 2014). Here we give a special emphasis on these works. We hope this review will give an overview of advanced physical gene delivery techniques for scholars and stimulate new ideas generated in gene therapy field.

## 2. Physical methods of gene delivery

The principles of physical methods are to use diverse physical forces such as electric, magnetic, ultrasonic and laser to deliver exogenous nucleic acids into cell. According to the physical forces, the principles of gene therapy are different, which are described in the following section.

### 2.1. Electroporation

**Figure 1.**
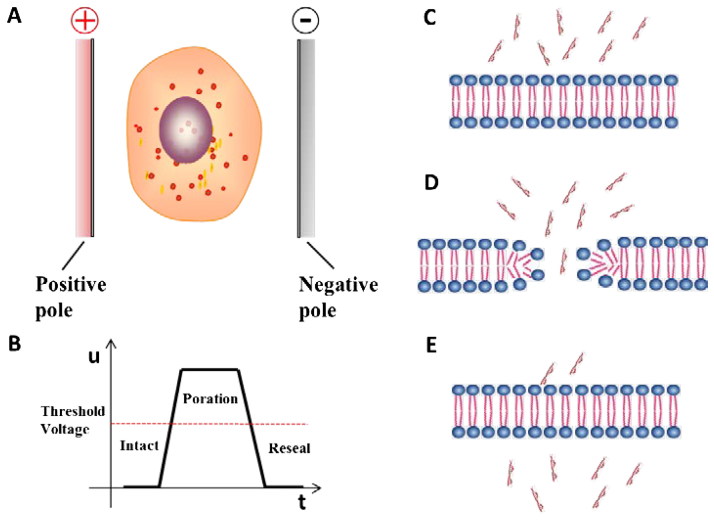
The principle of electroporation.

Utilizing a common physical tool electric field to change the permeability of cell membrane is called electroporation. It was firstly mentioned in 1982 by Neumann who employed electroporation to transfect mouse lyoma cells. The permeability of cell membrane has strong correlation with the intensity of external electric field. According to the exposure duration and electric field strength, the process of electroporation is divided into four continue phases including no detectable poration, reversible poration, non-thermal irreversible poration and thermal irreversible poration respectively (Yarmush et al., 2014; Megli & Kotnik, 2015). Gene therapy is performed in the phase of reversible poration (Miklavcic et al., 2014; Wagstaff et al., 2016), and the principle is shown in Figure 1. A lively cell is exposed in the external pulse electric field (Figure 1A). When the strength of external electric field exceeds the threshold voltage, the transient pore is formed in the cell membrane and the exogenous nucleic acids are delivered into the cell (Figure 1D). Then, the cell membrane resealing happens over a range of minutes (Figure 1E) after the strength of external field dropping down to the threshold voltage.

Electroporation has been used in in vivo gene transfer since 1991, and it has not been widely investigated until 1996 in many tissues, such as skin, liver, muscle and tumor (Suzuki et al., 1998; Miyazaki & Aihara, 1998; Vanbever & Préat, 1999; Bettan et al., 2000). Recently, many reports showed that electroporation were used for gene transfer *in vivo* (Wang et al., 2014; Latella et al., 2016; Bugeon et al., 2017; Ito et al., 2014). Wang et al. (2014) established an in vivo gene delivery system for injecting DNA vaccines based on electroporation. They utilized the minicircle DNA carrying a codon-optimized HIV-a gag gene to evaluate this system and found that electroporation further promoted the expression efficiency of minicircle gene. They demonstrated that the platform can been used to transfect DNA vaccines and increase the expression efficiency. Latella et.al (2016) employed the approach of electroporation to edit human mutant rhodopsin gene using plasmid-based CRISPR/Cas9 in the mouse retina, which demonstrated that CRISPR/Cas9 system was effective applied in vivo as a genetic engineering tool.

In the electroporation system, electrode plays an important role. By now, there are multiple types of electrodes have been designed including needle electrodes, plate electrodes, spoon electrodes and multielectrode arrays. Needle electrodes are needlelike electrode which could be inserted to varying depth. Excepting the electrode of two needles, multiple needle arrays were designed, such as six needles, which could change the polarity of the electric field to maximize cell permeability (Jaroszeski et al., 1997). Plate electrodes are surface-applied plate electrodes, which are placed on the skin by tweezers or calipers. The advantages of the plate electrodes are that the field between two electrodes is larger and uniform (Gehl et al., 1999). Like the multiple needle arrays, four-plated electrodes are designed by Heller, which is more effective, and the applied electric field can be rotated to enhance gene delivery. Spoon electrodes are based on electroporation cuvettes, on which vessel segment can be placed to electroporate. This electrode has been used to many samples with various sizes, such as rat carotid (3 mm), rat small intestine with a diameter up to 8 mm (Young & Dean, 2015). Another innovative electrode was multielectrode array which was designed by Heller for use in the skin (Ferraro et al., 2009; Heller et al., 2010; Guo et al., 2011; Amy et al., 2011). Heller and his teammates have devoted in the research of electroporation for a long time (Heller et al., 1996; Heller et al., 2000; Heller & Heller, 2006; Heller et al., 2008). Multielectrode array was designed to resolve the minor pain caused by electroporation, and made this approach more acceptable in clinical application. Recently, a new type of electrode is reported by Huang et al. They designed a planar electrode with advantage of minimally invasive, good biocompatibility, and lower applied voltage, and the procedure is depicted by Huang et al. in their article (Huang et al. 2017). Compared to the commercial device, this electrode has the advantage of higher transfection rate and less damage.

In despite of electroporation has been widely researched and applied, it is still limited by following drawbacks: (a) the transfection efficiency of electroporation is different depending on the tumor type; (b) cell viability of electroporation is still low.

### 2.2. Magnetoporation

**Figure 2.**
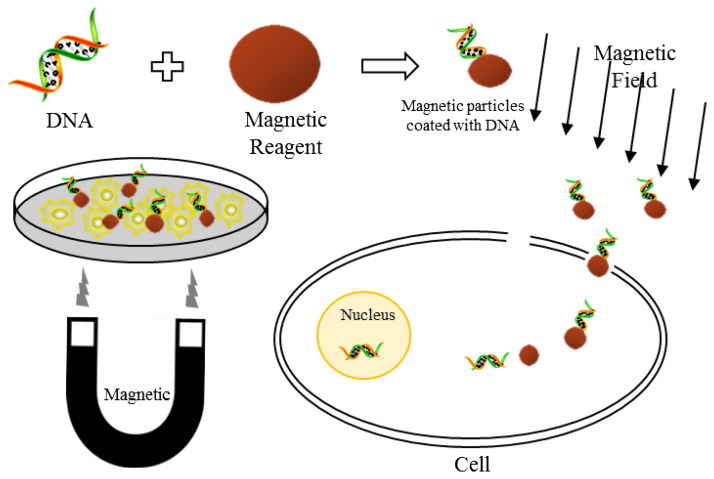
The principle of magnetoporation.

Magnetoporation is to deliver the nucleic acid into the cell under the influence of magnetic field, which is firstly proposed in 1996 with a form of patent literature (Chan, 1998) and is firstly published with a form of scientific literature in 2000 (Plank et al., 2000; Mah et al., 2000). Figure 2 shows the principle of magnetoporation. Exogenous nucleic acids are mixed with magnetofection reagent to form a biomolecule/magnetic reagent complex. Then the complex is delivered into cell in the force of magnetic field. Under the effect of magnetic field, the endocytosis and pinocytosis of cell membrane are accelerated (Arora et al., 2013; Das et al., 2015; Herrero et al., 2017). However, some scholars consider that the principle of magnetoporation is similar to electroporation. They deem that the magnetic field induces an electric field which changes the transmembrane potential of cell membrane. When the transmembrane potential reaches to a certain threshold, the cell membrane is perforated (Jakutavičiūtė et al., 2017).

Magnetofection reagents play an important role in this process, which bear the magnetic force and carry the nucleic acids into cell. The nucleic acids combine with magnetofection reagents by electrostatic interaction or salt-induced colloid aggregation (Mehier-humbert & Guy, 2005; Arora et al., 2013). The magnetofection reagents, such as CoFe_2_O_4_, NiFe_2_O_4_ and MnFe_2_O_4_, exhibit superior transfection efficiency than other magnetic materials (Sun et al., 2000; Tomitaka et al., 2010). However, these reagents are highly toxic, which limits their application both in vivo and in vitro (George et al., 2011; Cho et al., 2012). Iron oxides (Fe_3_O_4_, γ-Fe_2_O_3_) are commonly employed as magnetofection reagent due to its advantages of low toxicity and biocompatibility (Arora et al., 2013; Das et al., 2015). Sohrabijam et al. coated the iron oxide nanoparticles with chitosan and used it for magnetofection. They demonstrated that transfection efficiency was significantly increased and the particles were nontoxic (Sohrabijam et al., 2017). Shi and his teammates devoted to research the nanocomposite of iron oxide nanocrystals which were used as magnetofection reagent and showed better magnetofection efficiency (Shi et al., 2015; Shi et al., 2016). In addition, carbon nanotubes (CNTs) are also used as magnetofection reagent to enhance the transfection efficiency of magnetoporation (Cai et al., 2005; Liu et al., 2012).

The applications of magnetoporation in vitro and in vivo have been reported and shown better efficiency, such as cardiac tissue (Li et al., 2008), skeletal muscle (Zhou et al., 2007; Pereyra et al., 2016), liver tumors (Almstäetter et al., 2015), mouse myoblast (Akiyama et al., 2010), and mouse brain (Hashimoto & Hisano, 2011; Sapet et al., 2012; Soto-Sanchez et al., 2015). Moreover, magnetoporation is often used to transfect the difficult-to-transfect cells. Pereyra et al. (2016) recombined the adenoviral vectors with iron oxide nanoparticles into magneto-adenovectors to transfect the C2C12 myotubes in vitro and mouse skeletal muscle in vivo. Their results demonstrated that the magneto-adenovectors could improve the transfection rate of myotubes and enhance the transfection efficiency of muscle cells. Central nervous system is difficult to transfect under the effect of static magnetic field (Pickard et al., 2011). Therefore, Adams et al. (2013) employed the oscillating magnetic fields to transfect the neural stem cell, and over two-fold transfection efficiency was acquired. Cui and his teammates devoted to research transfection of animal cells through magnetofection (Wang et al., 2013; Y. Wang et al., 2014; Zhao et al., 2014; Chen et al., 2015). Recently, they reported a successful study on magnetoporation in plant transformation. Firstly, they introduced the exogenous DNA into the pollen under the effect of magnetic field. Then, the transfected seeds were successfully generated by pollination. Further, the exogenous DNA was successfully transferred into plant cell and expressed in the offspring (Zhao et al., 2017).

Although several applications have been reported in vitro and in vivo, the following drawback still is significant problem that restricted magnetoporation applying in clinic. That is the agglomeration of magnetofection reagents after removal of the magnetic field. Therefore, to explore new magnetofection reagent or resolve the phenomenon of cohesion still is a significant issue.

### 2.3. Sonoporation

Sonoporation is to perforate cell membrane by using ultrasound waves. Ultrasound frequency covers a broad range from 20 KHz to 5 MHz for gases and 500 MHz for liquids and solids (Mason, 1988), but it is attentive that sonoporation mainly uses ultrasound wave at megahertz frequencies (Mehier-humbert & Guy, 2005). Ultrasound was first used to transfect mammalian cell in vitro in 1996 (Kim et al., 1996) and was widely used for gene delivery in the 2000s (Miller & Song, 2003; Lu et al., 2003; Koike et al., 2005; Miao et al., 2005).

**Figure 3.**
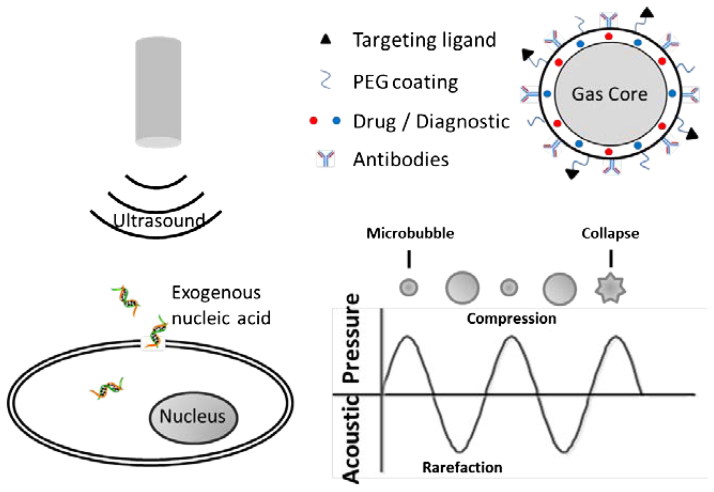
The principle of sonoporation.

Similar to electroporation, high intensity ultrasound field can also form a pore on the cell membrane. Then the exogenous nucleic acid is delivered into the cell. Two types of physical effects either thermal or non-thermal are produced when ultrasound acts on the cell membrane (Delalande et al., 2013). At low ultrasound intensity, non-thermal effects are generated including cavitation, mechanical streaming and radiation forces (Delalande et al., 2013). These effects are employed to perform ultrasound-mediated delivery. Cavitation is formed in a liquid that containing gaseous bubbles driven by a low intensity ultrasound (Zhou et al., 2014). With the ultrasound intensity changing with a sine shape, the cavitation bubble is periodic transforming between the states of compression and rarefaction. When the ultrasound intensity is instantaneously increasing, the cavitation bubble collapses immediately (Figure 3), which causes shock waves and microjets to perforate the cell membrane (Newman & Bettinger, 2007; Fan et al., 2014). However, sonoporation shows lower transfection efficiency than electroporation. In the last couple of years, microbubble is employed to improve the transfection efficiency of sonoporation. Microbubble is gas-filled vesicles encapsulated by stabling shell which can be functionalized by drugs, PEG, targeting ligands and antibodies (Dasgupta et al., 2016). The presence of microbubbles can reduce the threshold of sonoporation and prompt gene delivery (Tomizawa et al., 2013). However, the transfection efficiency has significant correlation with the concentration of microbubbles. Shapiro et al. (2016) reported that the concentration of microbubbles should be controlled in a tight range to achieve enhanced sonoporation. Too high or too low concentration of microbubbles can reduce the transfection efficiency of sonoporation. Recently, nanobubbles as an effective contrast agent are also used for sonoporation-mediated gene transfection by some scholar (Nishimura et al., 2017; Abdalkader et al., 2017). In addition, other nanocarriers, such as polymeric nanoparticles, polymeric micelles, liposomes or nanoemulsions, are also combined with sonoporation to enhance the transfection efficiency (Husseini & Pitt, 2008).

Currently, sonoporation has been used for different tissues, such as cardiovascular system (Unger et al., 2014), breast cancer (Bai et al., 2015), liver cancer (Zhang et al., 2016; Shen et al., 2016), pancreatic cancer (Kotopoulis et al., 2014), endothelial cells (Skachkov et al., 2014), and kidney tubules (Kurosaki et al., 2014). However, the transfection efficiency of sonoporation is subjected to several factors including the frequency and intensity of ultrasound wave, ultrasound pressure, and exposure duration (Mehier-humbert & Guy, 2005; Al-Dosari & Gao, 2009). Another factor is ultrasound contrast agent, which can lower the threshold of ultrasound cavitation and improve the transfection efficiency. But the cavitation is rarely precision controlled within the tissues. Therefore, improving the uniformity of cavitation and the accuracy of cell contrast could increase the transfection efficiency of sonoporation (Mellott et al., 2013).

### 2.4. Optoporation

**Figure 4.**
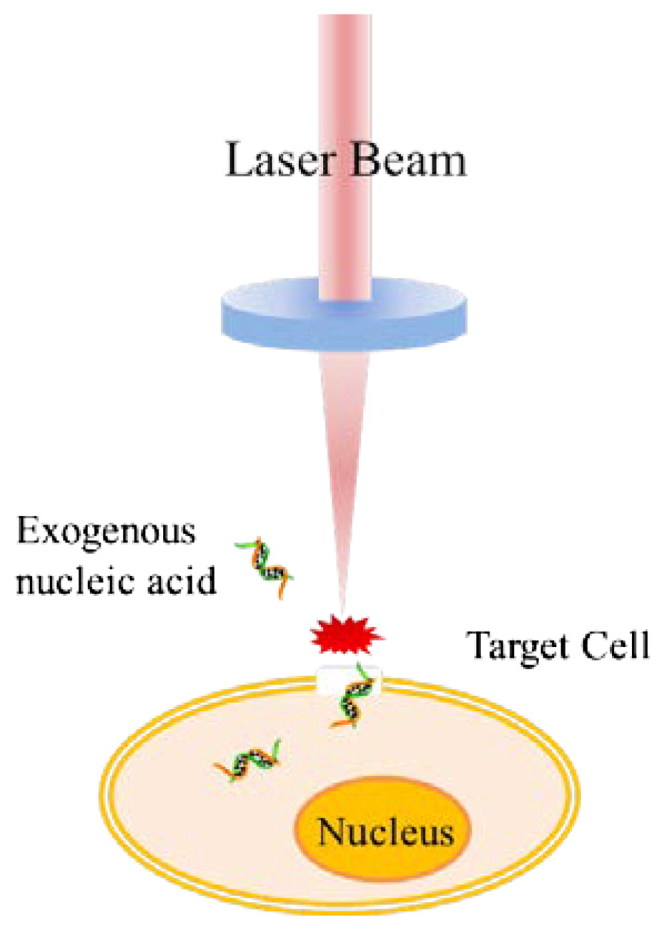
The principle of optoporation.

Optoporation is perforating the cell membrane with the help of laser beam. It was first reported to transfect normal rat kidney cell in 1984 by Tsukakoshi (Tsukakoshi et al., 1984). They used an ultraviolet nanosecond laser with 0.5 µm spot size and 1 mJ laser energy to punch cell and transfer the Ecogpt gene into the targeting cell. Their results proved that 10^3^ cells could be modified in one minute, tenfold transfection efficiency was acquired compared to the manual method, and the success rate was increased to three orders of magnitude compared to chemical method with a efficiency of at least one in 10^2^ cells. The principle of optoporation shows in Figure 4, a laser beam is focused on cell membrane to ablate the cell membrane, form a transient hole, and the exogenous nucleic acid was delivered into cell. High intensity laser irradiate the cell and produce plasma, which create high pressure and act on cell membrane to enhance the permeability of cell membrane (Noack & Vogel, 1995). Also, plasma can generate bubbles that ultimately collapse and produce secondary shock waves to perforate the cell membrane (Doukas & Flotte, 1996).

In our previous review, four different laser-assisted transfection techniques were introduced that included optoinjection, laser-induced stress waves, photochemical internalization, and irradiation of selective cell targeting with light-absorbing particles (Yao et al., 2008). The mechanism of laser-cell interaction is different depending on the type of laser. Until now, femtosecond laser, nanosecond laser, microsecond laser, and continuous wave laser are used for cell poration. However, each laser shows different perforating mechanism. Femtosecond laser with high repetition rates can create low density plasma to produce a single pore. With the increasing of laser repetition rates, pulse energies are enhanced and very small transient cavities are generated (Stevenson et al., 2010). Nanosecond laser can create cavitation bubbles and generate heating and thermoelastic stresses to perforate cell membrane (Stevenson et al., 2010). Therefore, femtosecond laser is extremely suitable for targeting single cell. Antkowiak et al. (2013) successfully transferred channelrhodopsin-2 into single selected neurons cell using femtosecond laser. In contrast, nanosecond laser is not suitable for single cell, because the size of pore produced by nanosecond laser is too large than single cell (Dijkink et al., 2008). Microsecond laser induce and create microbubbles, which creating shear stress to perforate cell membrane (Fan et al., 2014, 2015). Recently, Fan et.al reported to perforate single NIH/3T3 fibroblasts using microsecond laser (Fan et al., 2013). They focused microsecond laser pulse on an optically absorbent substrate, which creating a vapor microbubble. The microbubble oscillated in a fluidic chamber with a size of laser focal point. The shear stress following the bubble size oscillation can perforate the nearby cells. Their results showed that the cell transfection efficiency and cell viability was up to 95.2 ± 4.8% and 97.6 ± 2.4% respectively under the optimal poration conditions. With the continuous wave laser, the mechanism is to heat the cell membrane to enhance permeability (Stevenson et al., 2010; Fan et al., 2015).

The transfection efficiency is subject to energy intensity, pulse duration and the number of pulses (Mehier-humbert & Guy, 2005). Variety of laser wavelength can also influence the transfection efficiency. Lasers with various wavelengths have been used in optoporation including 193 nm, 308 nm, 355 nm, 405 nm, 488 nm, 532 nm, 800 nm, 1064 nm, 1554 nm and 2080 nm (Shirahata et al., 2001; Sagi et al., 2003; Badr et al., 2005; Paterson et al., 2005; Clark et al., 2006; Tsampoula et al., 2008; He et al., 2008; Yamaguchi et al., 2008). However, some components of tissues are strongly absorbing in visible region (Yao et al., 2008; Qin & Bischof, 2012). Therefore, near-infrared wavelengths, such as 800 nm or 1064 nm, are more suitable for optoporation in clinic application (Yao et al., 2008). Lei et al. (2008) employed a femtosecond laser micro-manipulation system with a central wavelength of 800 nm to perforate vital cells. Their results showed that the transfection efficiency of optoporated neurons can up to 91% for PC12 cells and 100% for astrocytes.

A few years ago, nanoparticle was employed to enhance the permeability of cell membrane for its merits that light can be absorbed, enhanced or scattered by nanoparticle. Umebayashi et.al described a laser-latex system that 532.5 nm Nd:YAG laser was employed to irradiate the mixture of latex particles and mouse fibrosarcoma (Meth-A) cells. They demonstrated that the proportion of permeabilized-resealed cells was affected by some factors, such as light intensity, irradiation time and particle concentration. Their results suggested that this research provided a new approach for delivering exogenous materials into living cells (Umebayashi et al., 2003). However, their report did not demonstrate the usefulness of this technique for macromolecules such as polypeptides, plasmid DNA. Schomaker et al. (2009) introduced the macromolecules (DNA) into cells using the technique of nanoparticle mediated laser perforation. Femtosecond laser and 150 nm gold particles were employed to perforate the cell membrane, and GFSHR-17 rat cells were successfully transfected.

Our laboratory has devoted in studying nanoparticle mediated optoporation for a long time (Yao et al., 2005a, 2005b; Hüttmann et al., 2005; Hüttmann et al., 2005; Yao et al., 2008; Yao et al., 2009a, 2009b; Yao et al., 2017). We demonstrated that laser irradiated nanoparticles can increase the permeability of cell membrane (Yao et al., 2005a, 2005b). We described a conjugate that nanoparticles were bounded to cell membrane by antibody, which can change the cell membrane permeability. 15 nm and 30 nm gold nanoparticles were respectively bonded to Hodgkin’s disease cell line L428 and human large-cell anaplastic lymphoma cell line Karpas 299 by the antibodies of BerH2 and ACT1. A Q-switched frequency doubled Nd: YAG laser at 532 nm was used to irradiate the conjugates. Under the optimal conditions, the transfection can up to 68% (Yao et al., 2005a). Furthermore, we demonstrated that the transfection efficiency and death ratio of cell were subject to laser parameters including pulse duration, irradiation mode, irradiation frequency, and irradiation manner (Yao et al., 2009a). In addition to laser parameters, the transfection efficiency also depends on gold nanoparticle (AuNP): cell ratio, cell-incubation medium, and cell-AuNP incubation time. In our recently publication, we functionalized AuNP by conjugation of antibody cetuximab against EGFR, and OVCAR-3 cells were incubated with AuNP-antibody conjugates. Then, we investigated the influence factors for cell membrane permeability in different experimental conditions. The results showed that cell permeability and viability were influenced by several factors including AuNP conjugation, AuNP concentration, irradiation fluence, cell condition, cell environment, and cell-incubation time with AuNP conjugates (Yao et al., 2017).

Recently, Bergeron et.al described an approach to perforate specific cell without affecting the surrounding cells. They functionalized the citrate-capped AuNP with orthopyridyl-disulfide-poly (ethylene glycol) (PEG) (5 kDa)-N-hydroxysuccinimide conjugated to monoclonal antibodies and HS-PEG (2 kDa or 5 kDa). Then, a near-infrared femtosecond laser was employed to perforate the AuNP attached cells. This method can selectively perforate specific cell without affecting the surrounding non-target cells (Bergeron et al., 2015). Although, gold nanoparticles were widely used for nanoparticle mediated optoporation, it is not free from several drawbacks that random adsorption of gold nanoparticles in the stage of preincubation. This stage can decrease the delivery efficiency and lower reproducibility of independent optoporation runs. Vanzha et al. (2017) proposed a new approach to solve this drawback. They immobilized gold nanostars on the culture plate and cells grown on it. Continues wave near infrared laser was employed to irradiate the cell. These proof-of-the-concept experiments demonstrated that the new approach can increase the permeability of propidium iodide into cell.

In addition to gold nanoparticles, carbon nanoparticles are also used for nanoparticle mediated optoporation. Prausnitz and his teammates reported that carbon black nanoparticles can facilitate the exogenous agents such as small molecules, protein and DNA into cells. Their results showed that more than 90% of DU145 prostate-cancer cells were successfully transfected at the optimal conditions, and the cell viability was more than 90%. Also, their results suggested that laser acted on carbon black nanoparticles may generate photoacoustic forces which cause transient poration on the cell membrane (Chakravarty et al., 2010). Different from their previous study, they employed nanosecond-pulsed laser to research the carbon black nanoparticle mediated optoporation. They found that transfection efficiency can up to 88% but no significant loss of cell viability at the conditions of lower fluence, lower concentration of carbon black nanoparticles and longer exposure times (Sengupta et al., 2014).

Optoporation is excellent to be used for single cell poration with high transfection efficiency and cell viability. However, it has lower transfection efficiency at targeting large populations of cells. In addition, the laser-microscope system is very expensive and huge. Therefore, cheap and portable laser-microscope system still is a research direction.

## 3. Conclusions and outlook

The main purpose of this article is to present four types of the advanced physical poration techniques which are used for gene transfection and drug delivery. Physical techniques can directly transfect cell without carrier vectors, and they are easy to prepare, possible to transfect large molecules, and safe to manipulate. However, ideal approach for cell poration is with high transfection efficacy and low toxicity. In all the physical techniques, electroporation is one of the most effective physical techniques, and has been widely used in vitro and in vivo. Unlike other techniques, electroporation can transfect a large number of cells in a short time. In addition, electroporation can transfect the primary cell types that are recalcitrant for lipid nanoparticles and non-viral transfection agents (Van Meirvenne et al., 2002). However, the cell viability of electroporation is unsatisfactory. With the magnetoporation, the transfection efficiency is lower than electroporation. It does not necessarily increase the transfection efficiency, indeed, it enhances the delivery speed with the help of magnetic transfection reagents (Plank et al., 2003). However, the magnetic transfection reagents may aggregate after vanishing of the magnetic field and lead to potential toxicity. Sonoporation also shows lesser transfection efficiency than electroporation. Therefore, ultrasound contrast agents are often employed to increase the transfection efficiency of sonoporation. A vital drawback of sonoporation is that it is difficult to precisely control. However, sonoporation is noninvasive as same as magnetoporation. Compared with other physical approaches, optoporation is less affected by the cell types and tissues. It is eminent to be used for single cell with high efficiency but poorly for large number population of cells.

In summary, as shown in Table 1, each technique has its advantages and disadvantages. Therefore, it is difficult to draft out a “league table” in terms of their transfection efficiencies. Thus, preferential use of one technique is depending on its characteristics and existed applications. For example, optoporation is a winner for single-cell poration, while electroporation and sonoporation are more suitable for perforating a large number population of cells. Neuronal stem cells are incredibly difficult to transfect, whereas, magnetoporation overcomes this problem (Sapet et al., 2011; Adams et al., 2013). Each of them has their own merits and drawbacks. Therefore, we can choose the most suitable approach depending on our experimental and clinic needs.

**Table 1.** Physical transfection approaches.

Physical techniques	Principle	Materials	Advantages	Disadvantages
Electroporation	Perforating cell membrane by electric field	Electrodes; Pulse generator	Simplicity; Lower cost; No need for vector	Invasiveness; Short-term pain; Tissue damage
Magnetoporation	Perforating cell membrane by magnetic field	Magnetic field; Magnetic transfection reagents	Noninvasive; Transfection reagents increase the efficiency	Lower efficiency with naked DNA; Transfection reagents aggregation
Sonoporation	Perforating cell membrane by ultrasound	Ultrasound probe; Ultrasound contrast agents	Noninvasive; Ultrasound contrast agents increase the efficiency	Lower precision; Lower Reproducibility; Tissue damage
Optoporation	Perforating cell membrane by laser pulse	Laser microscope system; Nanoparticles	Less dependent on cell type; Single-cell poration	Tissue damage; Low irradiation area; Low penetration capacity

Till now, there are many applications have been reported in in vivo and in vitro. However, only a few are applied in clinic (Boudreau et al., 2012; Kotopoulis et al., 2013; Weiland & Ahlén et al., 2013; Richard & Heller, 2015; Dimcevski et al., 2016). Most of them are in the stage of experiment, or are validated in animal. Therefore, aiming at the drawbacks of each technique, some endeavors are expected. For example, it is well known that the electrodes used in electroporation are invasive, thus, a type of mini-invasive electrode are necessary. Huang et al. (2017) designed a mini-invasive electrode, which had been used in in vivo. The next generation of electrodes should aim at noninvasive and friendly electrode. Magnetic transfection reagents may aggregate after vanishing of the magnetic field. Therefore, a new easily dissolved magnetic transfection reagent is expected for clinical application. Sonoporation is difficult to control the uniformity of cavitation, therefore, it cannot be controlled as precisely as electroporation. So, the next goal for sonoporation is to solve the control precision. Optoporation is precisely for targeting single-cell but with a lower throughput. Therefore, high throughput is the next direction for optoporation.

In summary, elucidating these advanced physical techniques aim to contribute to developing new delivery strategies with high efficiency, high cell viability and minimal risks. Therefore, the following endeavors are expected for these physical techniques. First, these physical forces may denature the proteins or surrounding tissues of targeting cells. Therefore, it is an important direction to precisely perforate the targeting cells with no damage for surrounding tissues. Second, transfection efficiency and cell viability should be improved. Compared with chemical method and biological method, the transfection efficiency of physical method is relatively lower. Although the transfection efficiency of electroporation is high, the low cell viability is a major drawback. So, high transfection efficiency and cell viability are the next direction to improve. Last, all of these physical techniques require a breakthrough for clinical applications.
